# Inducing the Production of Secondary Metabolites by Foliar Application of Methyl Jasmonate in Peppermint

**DOI:** 10.3390/plants12122339

**Published:** 2023-06-16

**Authors:** Wafae Kandoudi, Szilvia Tavaszi-Sárosi, Eva Németh-Zámboriné

**Affiliations:** Department of Medicinal and Aromatic Plants, Hungarian University of Agriculture and Life Sciences, Villányi St. 29-35, 1118 Budapest, Hungary

**Keywords:** elicitation, essential oil, mentha, phenolics, phytohormones

## Abstract

*Mentha x piperita* is a major source of secondary metabolites (SMs), and developing tools to enhance these compounds would be beneficial to meet the increasing demand in the industry. Elicitation by plant hormones became a new strategy to reach this goal. Three experiments in a climatic chamber and two experiments in an open field were conducted with peppermint to explore the effect of methyl jasmonate (MeJa) on the essential oil (EO) content, EO composition, and the total phenolic content (TPC). The treatment was applied for all experiments by spraying the aerial parts of the plants with a dosage of 2 mM MeJa twice. The treatment influenced all the parameters studied in the trials. The volatile content increased by 9–35%; however, in one trial it remained unchanged. The treatment also affected the main compounds of the EO. Menthone increased significantly in two trials while pulegone and menthofuran decreased. In the case of menthol, the change may also be influenced by the phenological and developmental stages of the plants. In the majority of cases, the TPC was also elevated considerably due to the treatments. MeJa treatments may have promising effects in influencing the accumulation of biologically active compounds and the quality of the drug; therefore, further systematic studies are needed to optimize the technology in vivo.

## 1. Introduction

Peppermint (*Mentha x piperita* L.) belongs to the *Mentha* genus in the Lamiaceae family, popular for its aromatic and medicinal properties. It is one of the most prominent cultivated species of mint. *M. piperita* is a perennial herb, a hybrid of *M. aquatica* and *M. spicata* [1]. It is a cosmopolitan plant and has significant uses in the cosmetic, pharmaceutical, food, and flavoring industries. Traditionally, peppermint leaves have been used against colds, skin problems, nausea, and diarrhea, along with other digestive and renal disorders [2].

Peppermint accumulates a variety of bioactive metabolites that are responsible for pharmacological and physiological effects such as antimicrobial, antioxidant, antispasmodic, and cardiovascular properties [3]. The distillation of the aerial parts produces peppermint essential oil (EO), a highly valuable product in the industry, composed mainly of monoterpenes such as (−)-menthol, with smaller amounts of its isomers, such as (+)-neomenthol and (+)-isomenthol, together with menthone, menthofuran, menthyl acetate, 1,8-cineole, limonene, neomenthol, and pulegone, etc. However, sesquiterpenes would only be present in smaller proportions with β-caryophyllene and germacrene D as the main compounds [4]. Peppermint extracts are also rich in phenolic compounds, such as eriocitrin, luteolin 7-O-glucoside, and rosmarinic acid, although, their distribution in the plant may vary significantly [5,6].

The production of secondary metabolites (SMs) can be affected by many factors, including genetic (subspecies, cultivars, chemotypes), morpho-phenological and environmental conditions (weather, soil, etc.) [7]. Although peppermint does not demonstrate a large chemical diversity, the composition, and thus, the quality of the drugs, depends on several factors. It has long been known that there are significant changes in the main volatile compounds of the shoots (menthol, menthone, and menthyl acetate) during ontogenesis [8]. With the appearance of the generative organs, the ratio of menthofuran and pulegone starts to elevate. Mcconkey et al. [9] declared that monoterpene production in peppermint may be a result of changes in the rate of biosynthesis during the development of leaf glandular trichomes and is restricted to leaves of 12 to 20 days of age. Several publications have also described that agrotechnological measures such as propagation methods or irrigation may influence—directly or indirectly—the accumulation and spectra of volatiles [7,10]. In some cases, mild drought stresses could stimulate the production of EO content [11,12]. Moreover, Abdi et al. [13] showed in their report that mild water deficit stress not only enhanced the EO production but also shifted the ratio of the major volatile compounds by significantly increasing the ratio of menthol and decreasing the menthone ratio.

With regard to the phenolic compounds of peppermint, fewer data have been published. It has been established that dry conditions stimulate their production. According to several reports, the total phenolic content (TPC) of peppermint samples seems to increase significantly in stressed plants [11,12,14,15]. Moreover, the ratio of the major phenolic compounds seems to increase remarkably, (such as rosmarinic acid and rutin compounds) [16].

The exposure of plants to biotic and abiotic stressors may lead to the activation of the defense mechanism and subsequently, the stimulation of the production of SMs [17]. Therefore, the application of any physical or chemical agents has become one of the prominent biotechnological tools to enhance the yield of SMs in vivo and in vitro, and it is referred to as “elicitation” [18,19]. Natural molecules produced by the plant but applied exogenously are also known as effective eliciting agents; however, until now this has primarily been under in vitro (bioreactor) conditions.

Jasmonates are widely known phytohormones and signaling compounds that are involved in the plant’s defense responses, as well as regulating several morphological and physiological processes influencing the tolerance of plants to both biotic and abiotic stresses [20,21]. The exogenous application of Jasmonates, particularly methyl jasmonate (MeJa), could enhance the accumulation of terpenoids and phenolics in some species [22]. Ali et al. [23] showed a significant increase in the production of TPC and total flavonoids in *Artemisia absinthium* L. cultures treated with 1 mg/L of MeJa 21 days before the measurements. Cell cultures of *M. piperita* under 100 µM MeJa also stimulated the accumulation of rosmarinic acid by 90%, 2 days post elicitation. However, in vivo elicitation comes with many challenges due to the heterogeneity of results and the scarcity of scientific evidence compared with in vitro elicitation. For instance, a treatment of MeJa (0.1 mM and 1 mM) applied once to *Agastache foeniculum* showed a significant elevation in the volatile production only with the lower dosage [24]. On the other hand, EO content was only increased in *Origanum majorana* L. with 2 mM MeJa, and the dosage of 0.1 mM did not influence the accumulation of volatiles [25]. Therefore, the current study aimed to shed light on the potential effects of in vivo application of MeJa on peppermint, one of the most widely cultivated medicinal and aromatic plants. A series of experiments, both under open field conditions and in a controlled environment, have been set up to reveal the reaction of peppermint to the above hormonal signaling and potential stressor molecule. Based on this, data may be obtained about the potential main influencing factors of the elicitation success. The ultimate goal of the study is the development of a simple, controllable, and reliable method for the improvement of peppermint drug quality.

## 2. Results

### 2.1. Essential Oil Content

**Figure 1:**
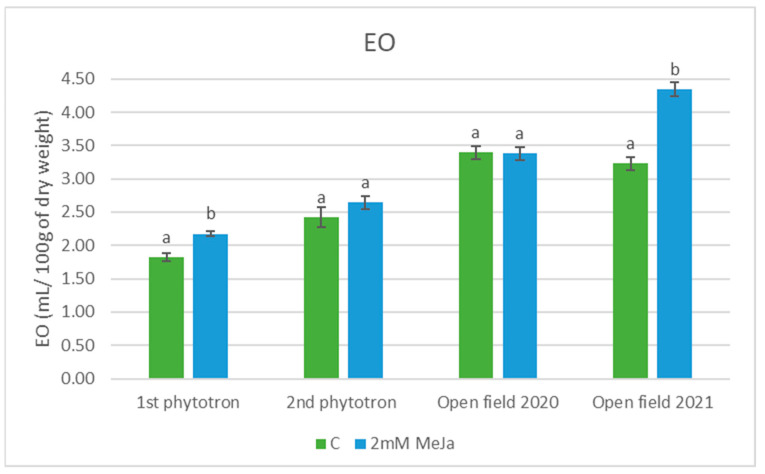
EO content of peppermint in four experiments. Data are mean ± SD (standard deviation); means with different letters are significantly different (*p* < 0.05).

The results shown in Figure 1 indicate that MeJa influenced the volatile accumulation of peppermint; however, this varied across the four experiments. The EO accumulation was enhanced by the MeJa treatment in all experiments (in two cases significantly), except for the first open field trial. In the first and second phytotron experiments, the increase was 19% and 9%, respectively, while in the second open field trial it reached 35%. In contrast, the EO content of the peppermint plants did not show any remarkable quantitative change in the 2020 open field trial. The differences could be statistically justified only in the first phytotron (*p* = 0.012) and second open field experiments (*p* < 0.01).

The treated samples in the 2021 open field experiment had the highest EO content of 4.372 mL/ mg of dry weight among all trials.

### 2.2. Essential Oil Composition

The results of the EO composition of peppermint plants are shown in Table 1 and Table 2. In the controlled environment experiment (2nd phytotron trial), the 2 mM MeJa treatment significantly increased the proportions of both of the two major components, menthone and menthol, by approximately 16%. In contrast, menthofuran, and its precursor, pulegone, were reduced by 10 and 40%, respectively, due to the treatment. Isomenthyl acetate was also affected by the treatment, while limonene and 1.8-cineole did not show any significant changes in the peppermint samples.

In the first open field experiment, the elicitation had no significant effect on the ratio of the evaluated major components. Only slight changes can be observed, such as a decrease of 5 and 8% in limonene and 1.8 cineole, respectively.

However, limonene, 1.8-cineole, and menthone were remarkably induced in the second open field trial by 38, 31, and 15%, respectively, due to the treatments. Menthol was reduced by 13%, along with isomenthyl acetate and pulegone, which exhibited the highest changes after exposure to MeJa (50% and 48% decrease, respectively). The remaining compounds, such as neomenthol and piperitone, did not show any significant shift in their ratios.

### 2.3. Total Phenolic Content

**Figure 2:**
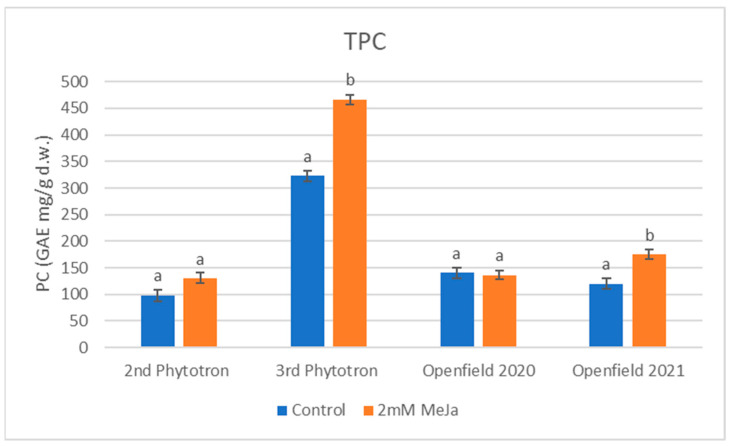
TPC of peppermint in four experiments. Data are mean ± SD (standard deviation); means with different letters are significantly different (*p* < 0.05).

The accumulation of phenolics in peppermint samples, determined as TPC, showed characteristic changes after MeJa treatments in all the experiments studied (Figure 2).

The open field 2021, second and third phytotron trials showed a prominent increase in this parameter by 46, 33, and 45%, respectively, among which the 3rd phytotron and the 2nd field experiments showed significance (*p* < 0.01). However, the TPC in the first open field experiment encountered a reduction, dropping from 140.51 mg GAE/g d.w. to 136.05 mg GAE/g d.w.

## 3. Discussion

With regard to the EO accumulation in peppermint, the effect of MeJa seems to be promising. In our trials, in most cases, elevations were produced due to the treatments. The biggest change was observed in the second open field experiment, where the volatile content increased to such an extent that it might even be economically significant in field practice. The first phytotron trial also resulted in a similarly beneficial response from the plant. These findings correspond to other investigations conducted on basil (*Ocimum basilicum*), coneflower (*Echinacea purpurea*), and spearmint (*Mentha spicata*) [26,27,28]. The enhancement of the EO content can be traced back to the fact that jasmonates are powerful signaling molecules involved in the metabolic processes of plants, including secondary metabolism [29,30].

No statistically significant changes occurred in the other two experiments, although slight increases and decreases in the contents were observed in the second phytotron and first open field experiments, respectively. A negative effect was also portrayed in a study on two species of thyme, *Thymus daenensis* and *Thymus vulgaris*, where two different dosages of MeJa (0.2 and 0.4 mM) applied three times in an open field decreased the EO yield [31]. At the same time, in another study, the same thyme species treated with 0.2 mM of MeJa once and grown in a controlled environment resulted in a rise in EO levels [32]. These findings support the statement that in vivo elicitation is not only dependent on the plant species and the dosage of the elicitor, but it could also be influenced by climatic conditions and the frequency of the treatments.

Our results confirm that MeJa may also alter the chemical composition of the volatiles in peppermint. Both in the first phytotron and the second field trials, a significant increase in the proportion of menthone and decreases in the ratios of its precursors, pulegone and the related menthofuran, were observed. Our results are supported by the findings of Soleymani et al. [33], who demonstrated that, after MeJa treatment, the transcript levels of the majority of the genes regulating the biosynthesis of these compounds gradually increased, and they reached the highest level at 72 h after treatment.

In conjunction with our findings, the enzymes responsible for the production of menthol and neomenthol showed no significant alterations. In our field experiment conducted in 2021, we observed decreased or unaltered levels of these particular compounds, as well as isomenthyl acetate, which is typically synthesized following them in the biosynthetic pathway. Thus, we may explain these compositional shifts by the involvement of MeJa in inducing the expression of genes responsible for the conversion of pulegone to menthone and subsequently menthol, but also downregulating the genes responsible for the formation of menthofuran. Nevertheless, the results may also be related to the phenological stage of the plants. In this experiment, the annual plants showed dynamic growth. With the rapid formation of new leaves, new essential oil glands were also developing, and this is where the biosynthesis of volatile molecules takes place intensively [34]. Therefore, in the harvested samples, these processes reflect a typical “early phase” EO composition [8].

In contrast to the previously mentioned results, the initial open field experiment conducted in 2020 indicated that MeJa did not have a significant impact on the EO composition of peppermint. The different responses between the two experiments could be explained by the age and condition of this plant stand. As mentioned above, the plantation used in that year was a 4-year-old plantation with relatively weak growth, and the plants had already developed flowers at a height of 30–40 cm. Thus, contrary to the 2021 population, there was minimal noticeable development of new leaves and EO synthesis during the two-week period from the second treatment until the harvest. As a result, the elicitor’s influence remained practically ineffective during that time frame.

The composition of the open field and the phytotron-grown plants also showed characteristic differences, as in the latter experiment much higher ratios of the compounds pulegone and menthofuran have been found compared with the first experiment. These differences are, however, probably not in direct connection with our treatments, but may be explained by the phenological stage of the plants. In the climatic chamber, the plants were harvested before budding in the vegetative stage; thus, the composition reflected a more typical unripe oil [8]. The elevated levels of the two components mentioned might also be the result of the limited light condition in the phytotron (16,000 W/m^2^ illumination) compared with the direct sunshine in the open field, as was demonstrated by Rios-Estepa et al. [35].

Soleymani et al. [33] concluded that MeJa might improve the medicinal properties of *M. piperita*. Based on the present trials, we may affirm the aforementioned statement. Our results indicate that MeJa may be a potential strategy for improving the quality of peppermint EO. Regulation of the proportions of menthol and menthone should, however, be carefully optimized to take into consideration the growing conditions, the timing of the treatment, and the harvest. In addition, the potential decrease of the ratios of menthofuran and pulegone may be of interest, as their percentages are restricted in foods and flavoring products due to the potential risk of hepatotoxicity [36].

Polyphenols are common valuable compounds in medicinal plants which have a wide range of biological properties [37]. Kim et al. and Złotek et al. [38,39] reported that MeJa/JA remarkably enhanced the accumulation of TPC in basil and butter lettuce (*Lactuca sativa* L.). Moreover, peppermint-elicited leaves with MeJa showed higher levels of phenolics [40,41]. These findings align with our results; the application of the phytohormone in both phytotrons and the second open field trial stimulated the secretion of the phenolic compounds. The last phytotron trial in 2021 exhibited values that were more than double, which can be attributed to the intense purple coloration of the plants caused by anthocyanin accumulation. Nevertheless, it is worth noting that the overall trend resulting from our treatment remained consistent across the different trials. The only exception was observed in the initial open field trial, where there was a slight decrease of 3% in TPC, although this change was not considered statistically significant. Interestingly, similar findings have been reported in the literature, where MeJa application on broccoli sprouts (*Brassica oleracea* L., var. *italica*) resulted in a decrease in TPC [42,43]. However, in this case, the old plantation with relatively weak development might also have contributed to the fact that the reaction of the plants was not intensive in this case. It seems that the elicitor may induce stronger stress responses and, as a result, a more significant stimulation of volatiles and phenolics in optimally developing plants.

## 4. Materials and Methods

### 4.1. Experimental Sites

The current study was carried out in two different experimental sites: two open field trials in the Experimental Station of the University of Agricultural and Life Sciences (MATE) in two consecutive years (2020–2021), in Budapest, Hungary (47.398820, 19.149270), and three controlled environment experiments in a phytotron chamber in MATE, Buda campus.

**Figure 3:**
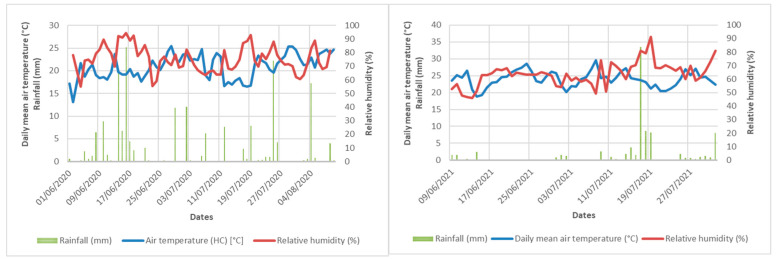
Daily mean air temperature (°C), rainfall (mm), and relative humidity (%) of the experimental field in the summer of 2020 and 2021.

The open field experiments were carried out in 2020 in a 4-year-old population and in 2021 in an annual population. The plantations represented the stock plantation of ‘Mexican’ cultivar (*Mentha piperita* L.) maintained by vegetative propagation in the MATE gene bank (accession nr. HUNGB401-LAMIMENT17) (Table 3. In the older plantation, row distances were no longer available; in the new plantation the row distance was 70 cm. The average temperature, precipitations, and air humidity data in the experimental field in the years 2020 and 2021 are summarized in Figure 3. In the dry periods, regular sprinkling irrigation was applied on the field.

For the phytotron trials, 12 cm long healthy stolons from the above-mentioned MATE mother plantation were transplanted into individual pots (12 cm diameter). The first experiment in 2020 served as a preliminary trial to measure the EO content; the second, also in 2020, was to determine the EO content, its composition, and the TPC, while the third experiment in 2022 was set to measure the TPC. The plants in all trials were grown in a climatic chamber (Fitotron SGC120, Weiss Gallenkamp Ltd., Loughborough, Leicestershire, United Kingdom) under the same conditions, with temperatures between 25 and 27 °C during the day and 15–17 °C at night. The light cycle was regulated at 14 h of light and 10 h of dark, and the air humidity was fixed at 65% for all experiments. The pots received a uniform and regular irrigation on every second day. The main properties of the soils at the experimental plots and in the climatic chamber are summarized in Table 4. The experimental plots represent sandy loam soil with relatively low humus content, while an artificial soil mixture with higher nutrient and humus content was used in the in vitro experiments (Florasca B) (Florasca Kft, Osli, Hungary).

### 4.2. Treatments

A completely randomized block design was arranged for the field experiment in three replications with a plot size of 2 m^2^. In the phytotron experiments, ten uniformly developed individual plants in pots represented each of the treated and non-treated groups and the sampling was carried out in three replicates, similar to the field experiment.

The treatment started at the budding stage and vegetative stage, respectively, for open field and phytotron trials. The plants were treated with 2 mM of MeJa (Sigma-Aldrich, Schnelldorf, Germany) in all current experiments. This dosage was chosen based on previous experience and other trials [25,44]. MeJa was dissolved in water mixed with 0.3% ethanol. The solution was foliarly sprayed using a hand pump sprayer which distributed the solution uniformly onto the leaves. Approximately 50 mL solution per plant was added in the open field, and 20 mL in the controlled environment. The control groups were sprayed at the same time with only water and ethanol. Two treatments were applied, repeating the first one after a week (Table 3).

### 4.3. Harvesting

A week after the second treatment, the above-ground parts of the plants were harvested by cutting the plants at approximately 10 cm (open field) and 5 cm (phytotron) above the soil surface. In the open field, at this time the plants were at the beginning of flowering, while in the phytotron, they remained still in the vegetative phase. The samples were air-dried in shade under ambient temperature. The treatment and the harvesting conditions were similar in all experiments.

### 4.4. Essential Oil Extraction

After drying for two weeks, the samples were taken to the laboratory where the leaves were separated from the stem parts, and they were used in three replicates for EO distillation; 20 g of dried material from each sample was hydro-distilled in a Clevenger-type apparatus using 500 mL of water for 1.5 h as suggested by the VII Hungarian Pharmacopoeia [45]. The oils were collected, and all traces of water were eliminated by anhydrous sodium sulfate, then stored in a sealed vial in a refrigerator at 4 °C before being analyzed. The EO content was expressed by mL/100 g of dry matter. The dry matter content was determined from the weight loss after heating the drug at 105 °C for 3 h in a thermostat.

### 4.5. Essential Oil Composition

The analysis of the chemical constituents of the EO was determined by GC–MS using an Agilent Technologies 6890N instrument equipped with HP–5MS capillary column (5% phenyl, 95% dimethyl polysiloxane, length: 30 m, film thickness: 0.25 mm i.d. × 0.25 µm) and an Agilent Technologies MS 5975 inert mass selective detector, both supplied by Agilent Technologies International Sàrl (Rolle, Switzerland). We used helium as a carrier gas (1 mL/min). The temperature during the identification of the components was scheduled at 60 °C initially, then increased by a rate of 3 °C/min up to 240 °C, with the final temperature being held for 5 min. The injector and detector temperatures were 250 °C and the split ratio was 30:1. An amount of 10 µL of EO was diluted with n-hexane to 1 mL and the injected quantity was 0.2 µL. The MS was recorded at 70 eV in full scan mode, which revealed the total ion current (TIC) chromatograms (mass range m/z 50–500 µma). The identification of the EO components was based on the comparison of their linear retention indices, which were calculated using the Van Den Dool and Kratz generalized equation [46], with the literature data, and by matching recorded mass spectra with those in the mass spectral library references (NIST MS Search 2.0 library, Wiley 275) and a mass spectra library [47].

### 4.6. Total Phenolic Content

A 24 h maceration was needed for the determination of the TPC. A quantity of 1 g of powdered dried plant material was obtained by grinding the dry leaves and adding them to 100 mL of boiling distilled water (used as a solvent), which was extracted after a day. Finally, the extracts were filtered and stored frozen awaiting further analysis.

The quantification of total phenolic content was determined by the modified method of Singleton and Rossi [48]. An amount of 0.5 mL of the sample solution was placed in a test tube with 2.5 mL Folin–Ciocalteau’s reagent (10 *v*/*v*%). After 1 min of incubation, 2 mL of sodium carbonate (700 mM) was added. The absorbance was measured at 760 nm in a Thermo Evolution 201 spectrophotometer (Unicam Magyarország Kft., Budapest, Hungary) after a 5 min incubation period in hot water (50 °C). For calibration, 0.3 M of gallic acid was used as the chemical standard. The total phenolic content of the sample was expressed as mg of gallic acid equivalents per g of the dry weight of extract (GAE mg·g^−1^ d.w.). A blank was prepared which contained distilled water instead of extract. The measurements were carried out in three replications. All reagents used were purchased from Sigma-Aldrich.

### 4.7. Statistical Analysis

Data were evaluated using means, standard deviations, one-way analysis of variance for EO content and TPC, and multivariate analysis of variance for EO composition, using IBM SPSS Statistics 25. A *p*-value less than 0.05 was considered statistically significant [49,50].

## 5. Conclusions

The application of elicitors represents one of the leading alternatives to increasing the SMs in medicinal and aromatic plants, although until now reliable results have been mostly accumulated under in vitro conditions. Based on the recent trials on peppermint, it is plausible to suggest that treatments with MeJa could potentially yield promising outcomes in terms of influencing the accumulation of biologically active compounds, thus enhancing the drug’s quality under cultivation circumstances. In three of the four evaluated trials, the volatile content increased; in two of them the increase was significant. Changes in the ratio of menthol were not consistent, while the ratio of menthone increased in two trials. Decreases in the ratio of menthofuran and pulegone might be considered as advantageous. The second open field stand that experienced the most rapid growth exhibited the most notable changes in volatiles and TPC. Additionally, there appears to be a correlation between the effectiveness of the elicitation process and the growth intensity of the plants.

These data suggest that the success of elicitation of peppermint in vivo may be the result of complex developmental and regulatory processes in the plant and influenced not only by the type and dosage of the elicitor but also by the plant’s phenological and physiological stage. Thus, all these factors must be taken into consideration and adjusted during optimization of the treatments.

Consequently, further systematic studies are required to understand these connections. Sampling of young and more senescent parts of the plants separately and monitoring the gene expression level of the relevant biosynthetic enzymes may lead to a more comprehensive understanding in the future.

## Figures and Tables

**Table 1:** The chemical composition of the peppermint EO from the second phytotron experiment.

Components	RI ^1^	Control	2 mM MeJa
Limonene	1029	2.27 ^a^	2.32 ^a^
1.8-cineole	1034	2.30 ^a^	2.60 ^a^
Menthone	1158	37.30 ^a^	43.27 ^b^
Menthofuran	1167	18.21 ^b^	16.36 ^a^
Menthol	1171	14.04 ^a^	16.33 ^b^
Pulegone	1236	9.18 ^b^	5.43 ^a^
Piperitone	1249	1.00 ^b^	0.76 ^a^
Isomenthyl acetate	1291	9.54 ^b^	7.54 ^a^
Total		93.84	94.61

^1^ Retention indices. Values within rows with the same letters (a,b) were not significantly different (significance level at 5%). Compounds which reached at least 1% are shown.

**Table 2:** The chemical composition of the EO of open field peppermint.

		Open Field 2020		Open Field 2021	
Components	RI ^1^	Control	2 mM MeJa	Control	2 mM MeJa
Limonene	1029	5.51 ^a^	5.23 ^a^	4.61 ^a^	6.38 ^b^
1.8-cineole	1034	4.28 ^a^	3.94 ^a^	5.50 ^a^	7.22 ^b^
Menthone	1158	35.05 ^a^	35.15 ^a^	30.06 ^a^	34.59 ^b^
Neo-menthol	1159	-	-	6.86 ^a^	6.11 ^a^
Menthofuran	1167	7.70 ^a^	7.91 ^a^	-	-
Menthol	1171	27.79 ^a^	27.73 ^a^	35.10 ^b^	30.30 ^a^
Pulegone	1236	1.96 ^a^	2.06 ^a^	0.82 ^b^	0.43 ^a^
Piperitone	1249	1.57 ^a^	1.66 ^a^	2.05 ^b^	2.09 ^a^
Isomenthyl acetate	1291	4.57 ^a^	4.12 ^a^	7.45 ^b^	3.85 ^a^
Total		91.21	90.60	94.92	93.65

^1^ Retention indices. Values within rows with the same letters (a,b) were not significantly different (significance level at 5%). Compounds which reached at least 1% are shown.

**Table 3:** Timeline of planting, treatment, and harvest.

	Propagation	1st Treatment	2nd Treatment	Harvest
1st phytotron experiment	23 January 2020	25 February 2020	3 March 2020	11 March 2020
2nd phytotron experiment	16 September 2020	5 November 2020	12 November 2020	19 November 2020
3rd phytotron experiment	2 November 2021	21 January 2022	28 January 2022	3 February 2022
1st open field experiment	Perennial plantation	26 June 2020	2 July 2020	10 July 2020
2nd open field experiment	30 March 2021	28 June 2021	5 July 2021	12 July 2021

**Table 4:** The soil composition of the experimental plot in 2020 and 2021.

Measured Parameter	pH H_2_O	Humus Content %	Lime Content%	Texture	NO_2_^+^NO_3_^−^N mg/kg	P_2_O_5_ mg/kg	K_2_O mg/kg	Zn mg/kg	Mg mg/kg	Mn mg/kg
Soil in the experimental station 2020	7.82	2.84	0.34	sandy loam	6.93	412.89	245.54	4.09	131.78	25.64
Soil in the experimental station 2021	7.47	1.62	4.20	sandy	16.70	398.67	826.33	8.08	174.19	138.99
Soil in the phytotron pots	5.49	8.16	<0.20	clay loam	1401.50	875.50	3357.40	12.40	829.00	54.70

## Data Availability

The data presented in this study are available within the article.
